# ﻿Advances in the taxonomy and distribution of *Scolomus* (Hymenoptera, Ichneumonidae), including the description of a new Andean species and an updated identification key

**DOI:** 10.3897/zookeys.1234.145472

**Published:** 2025-04-10

**Authors:** Rodrigo O. Araujo, Isamara Silva-Santos, Andrés Moreira-Muñoz, Cristian Montalva, Diego G. Pádua

**Affiliations:** 1 Laboratorio de Entomología General y Aplicada, Centro de Investigación de Estudios Avanzados del Maule, Universidad Católica del Maule, Avenida San Miguel, 3605, Talca, Chile Universidad Católica del Maule Talca Chile; 2 Programa de Pós-Graduação em Entomologia, Instituto Nacional de Pesquisas da Amazônia, Manaus, Amazonas, Brazil Instituto Nacional de Pesquisas da Amazônia Manaus Brazil; 3 Instituto de Geografía, Pontificia Universidad Católica de Valparaíso, Avenida Brasil, 2241, Valparaíso 2340025, Chile Pontificia Universidad Católica de Valparaíso Valparaíso Chile; 4 Laboratorio de Salud de Bosques, Instituto de Conservación, Biodiversidad y Territorio, Facultad de Ciencias Forestales y Recursos Naturales, Universidad Austral de Chile, Valdivia, Chile Universidad Austral de Chile Valdivia Chile

**Keywords:** Andean Region, Chile, Darwin wasps, Maulean Coastal Forest, parasitoid wasps, South America

## Abstract

*Scolomus* Townes & Townes, 1950 is a genus in Ichneumonidae, with six species occurring in the Holarctic and Neotropical regions. In this study, a new species is described from Chile, *Scolomusvalenzuelai* Araujo, Pádua & Silva-Santos, **sp. nov.** Also, the female of *S.magellanicus* Walkley, 1962 is described for the first time and new occurrences of *S.maculatus* Araujo & Vivallo, 2018, and *S.magellanicus* are reported from Chile, including the northernmost record of this genus in South America. Additionally, we provide an updated identification key for all known species of the genus.

## ﻿Introduction

Metopiinae Förster, 1869 is widely distributed and comprises 26 genera and more than 860 species (Yu et al. 2016; Ranjith and Priyadarsanan 2022). Members of this subfamily are koinobiont endoparasitoids that oviposit in lepidopteran larvae and emerge from the host pupa as adults (Broad and Shaw 2005; Bennett 2008). They can be distinguished by their strongly convex face, broad pronotum, relatively thick antennae, and shortened tarsal segments, which are adaptations believed to facilitate movement through semi-resistant substrates, such as partially silken host retreats or rolled-up leaves (Broad and Shaw 2005).

*Scolomus* Townes & Townes, 1950, is a small, widely distributed genus within Metopiinae, comprising six species: *S.borealis* (Townes, 1971), *S.clypeatus* Araujo & Santos, 2018, *S.maculatus* Araujo & Vivallo, 2018, *S.magellanicus* Walkley, 1962, *S.talamanca* (Gauld & Sithole, 2002), and *S.viridis* Townes & Townes, 1950. Little is known about the biology of this genus, except for a record of *S.borealis* as a parasitoid of the immature stages of *Schreckensteiniafestaliella* (Hübner, 1819) (Lepidoptera, Schreckensteiniidae) (Broad and Shaw 2005).

The genus was initially described in Tryphoninae (Townes and Townes 1949) but was later transferred to Pionini (Ctenopelmatinae) without justification (Townes and Townes 1966). Subsequently, Gauld and Wahl (2006) synonymized *Apolophus* Townes, 1917 under *Scolomus* based on morphological similarities. *Apolophus* and *Scolomus* exhibit several shared apomorphic features, including an elongated head with an extended malar space, a flat face with a weak supraclypeal suture, and a hind wing with a strongly curved basal abscissa of vein M+Cu1, where the distal abscissa of vein Cu1 joins vein cu-a closer to vein 1A than to vein M. Based on these characteristics, *Scolomus* was placed in Metopiinae, although its definitive position requires a detailed phylogenetic analysis of the Ophioniformes clade (sensu Wahl 1991, 1993) (Broad and Shaw 2005; Bennett et al. 2019).

In this study, we describe a new species of *Scolomus* from Chile and provide the first taxonomic description of the female of *S.magellanicus*. Additionally, we expand the knowledge about distribution range of both *S.magellanicus* and *S.maculatus*. As the new species rendered the initial steps of the identification key proposed by Araujo et al. (2018) unfeasible, we provide an adapted identification key for all known species of the genus.

## ﻿Materials and methods

The specimens were studied from the following institutions (curators in parentheses): **LEGA-UCM**: Laboratorio de Entomología General y Aplicada, Universidad Católica del Maule, Chile (Rodrigo Araujo); **MNNC**: Museo Nacional de Historia Natural, Chile (Mario Elgueta); **NMNH**: National Museum of Natural History, USA (Sean Brady); **UACh**: Colección de Insectos Ernesto Krahmer, Universidad Austral de Chile, Chile (Cristian Montalva).

The holotype of *Scolomusmaculatus* (MNNC) was examined, while the following holotypes were studied through high-resolution photographs: *S.magellanicus* (NMNH), *S.viridis* (NMNH), and *S.clypeatus* (lost). Regarding the last species, the holotype was destroyed in the fire that consumed the Museu Nacional do Rio de Janeiro, Brazil, and we relied on the photographs provided by Araujo et al. (2018), which we consider sufficiently detailed for study. The holotype of *S.valenzuelai* Araujo, Pádua & Silva-Santos, sp. nov. is deposited in the MNNC.

The type specimen of the new species was recently collected within remnants of the coastal Maulino forest (Escobedo et al. 2024), an endemic vegetation formation dominated by deciduous species such as *Nothofagusglauca* (Phil.) Krasser (Nothofagaceae). This forest type is highly threatened due to the historical replacement of native vegetation with *Pinusradiata* (D. Don) and *Eucalyptusglobulus* Labill plantations. The most hygrophilous remnants are characterized by the presence of Myrtaceae species, like *Lumaapiculata* (DC.) Burret and *Myrceugeniaexsucca* (DC.) O. Berg., alongside iconic Chilean trees such as *Drimyswinteri* J.R. Forst. & G. Forst. and *Persealingue* Nees. The understory is rich in native ferns and shrubs, notably *Blechnumchilense* (Kaulf.) Mett. (Doll et al. 2024).

General morphological terminology follows Broad et al. (2018) and proportions follow Araujo et al. (2018). The topics “type material” and “examined material” include the details provided on the label. The use of an asterisk (*) indicates a new distribution record.

Images were captured with a Leica S9i stereomicroscope with an LED illumination dome (Kawada and Buffington 2016). Measurements and image stacking were done using the Leica Application Suite X extended-focus software, followed by editing in Adobe Photoshop 2020. All measurements were rounded to the nearest 0.05 mm.

Distribution data for *Scolomus* spp. were extracted directly from specimen labels and plotted on a map using SimpleMappr (Shorthouse 2010).

## ﻿Results

### ﻿Taxonomy

#### ﻿ Scolomus

Taxon classificationAnimaliaHymenopteraIchneumonidae

Townes & Townes, 1950

F20F79E3-B1CD-59C3-BECE-0A49CB703277

Scolomus Townes and Townes 1950: 420. Type species: Scolomusviridis Townes & Townes, 1950, by original designation.
Apolophus Townes 1971: 111. Type species: Apolophusborealis Townes, 1971, by original designation.

##### Diagnosis.

The head is elongate, with an exceptionally long malar space measuring 1.20–1.80× the basal mandibular width. The clypeus is large, subquadrate, and with the clypeal sulcus weakly impressed or absent, which results in the face and clypeus forming a nearly uniform, smooth plane in most species. The occipital carina is ventrally incomplete. The mandible is slender, with the lower tooth 0.50–1.00× the length of the upper tooth. The fore wing features a rhomboid to pentagonal areolet (areolet lightly petiolate in *S.valenzuelai* sp. nov.), and the pterostigma is broad and triangular, with a maximum length 2.30–3.00× its maximum width. In the hind wing, the basal abscissa of vein M+Cu1 is strongly arched, and the distal abscissa of vein Cu1 connects to vein cu-a much closer to vein 1A than to vein M. The first metasomal tergite exhibits an anterior median depression bordered laterally by raised edges. The glymma is deep, converging at the midline, and often separated only by a translucent partition. In females, the hypopygium is large and arched but not folded medioventrally. The ovipositor is slender, slightly upcurved, and lacks a dorsal subapical notch.

##### Distribution.

The genus is distributed in the Holarctic region (Austria, England, Germany, Poland, Russia, Ukraine, and the United States of America) and Neotropical region (Argentina, Chile, and Costa Rica), which includes the Andean biogeographic zone (sensu Morrone 2015).

##### Included species.

*S.borealis* (Townes, 1971) (Nearctic, West Palearctic); *S.clypeatus* Araujo & Santos, 2018 (Andean); *S.maculatus* Araujo & Vivallo, 2018 (Andean); *S.magellanicus* Walkley, 1962 (Andean); *S.talamanca* (Gauld & Sithole, 2002) (Neotropical); *S.viridis* Townes & Townes, 1950 (Andean).

### ﻿Key to the world species of *Scolomus*

**Table:** 

1	Mandible stout, 3.00–4.00× as long as basal width. Labrum apex always visible, even with mandibles closed. Subtegular ridge produced as a sharp, curved spine (Figs 1–4). Deep groove between the propodeum and metanotum	**2**
–	Mandible slender, 5.50–6.50× as long as basal width; labrum more or less concealed when mandibles closed (Araujo et al. 2018: figs 1, 3, 5). Subtegular ridge not produced into a sharp spine. Superficial groove between the propodeum and metanotum	**4**
2(1)	Wings with a lightly petiolate areolet, rhombic, with vein 2rs-m joining 3rs-m shortly before touching RS (Fig. 3). Postscutellum rounded posteriorly (Fig. 2). Lateromedian longitudinal carina slightly sinuous, lateromedian longitudinal carinae almost parallel. Area basalis trapezoidal (Fig. 2). Wings strongly and entirely infuscate, pterostigma and all veins dark brown (Fig. 3)	Scolomusvalenzuelai **Araujo, Pádua & Silva-Santos, sp. nov.**
–	Wings with a pentagonal areolet, with vein 2rs-m complete and 3rs-m partially complete or faintly impressed, both touching RS independently (Fig. 6). Postscutellum straight posteriorly (Fig. 5). Lateromedian longitudinal carina oblique until the intersection with anterior transverse carina, converging or not. Area basalis triangular (Fig. 5). Wings hyaline to lightly infuscate, pterostigma and all veins brown (Fig. 6)	**3**
3(2)	Propodeum with lateromedian longitudinal carina defined until the intersection with anterior transverse carina and faintly impressed posteriorly. Area superomedia absent (Fig. 5). Lateral longitudinal carina sharper and more distinct near propodeal spiracle. Vein 3rs-m partially complete (faintly impressed only when touching vein M)	***S.magellanicus* Walkley, 1962**
–	Propodeum with posterior transverse carina strong and complete. Area superomedia present (although open posteriorly). Lateral longitudinal carina uniform throughout its entire length. Vein 3rs-m faintly impressed	***S.viridis* Townes & Townes, 1950**
4(1)	Propodeum with no trace of lateromedian longitudinal carina. Head and mesoscutum with reddish-brown marks, fore leg orange-brown. Central America	***S.talamanca* (Gauld & Sithole, 2002)**
–	Propodeum with lateromedian longitudinal carina discernible. Head and mesoscutum without reddish-brown marks, fore legs brown or green	**5**
5(4)	Propodeum with lateral longitudinal carina incomplete. Clypeus with simple, uniformly distributed setae in femaele. Body mostly blackish, without extensive green areas; Holarctic	***S.borealis* (Townes, 1971)**
–	Propodeum with lateral longitudinal carina complete. Clypeus with clusters of seemingly bifurcate setae in female. Body with extensive green areas. Chile	**6**
6(5)	Clypeus width 3.00× its height (Araujo et al. 2018: fig. 1). Distance between eye and lateral ocellus 1.50× diameter of lateral ocellus. Areolet 0.80× as wide as long. Hypopygium 1.55× as long as wide, in lateral view. Ovipositor 12.50× as long as basal width. Head, mesoscutum, postscutellum, and pronotum entirely brownish yellow	***S.clypeatus* Araujo & Santos, 2018**
–	Clypeus width 1.55–2.30× its height (Araujo et al. 2018: figs 3, 5). Distance between eye and lateral ocellus 0.90–1.00× diameter of lateral ocellus. Areolet 1.00× as wide as long; hypopygium 1.90× as long as wide, in lateral view. Ovipositor 5.30× as long as basal width. Head and pronotum greenish, mesoscutum yellow with dark brown spots on lateral lobes and around scutellum	***S.maculatus* Araujo & Vivallo, 2018**

#### ﻿ Scolomus valenzuelai

Taxon classificationAnimaliaHymenopteraIchneumonidae

Araujo, Pádua & Silva-Santos sp. nov.

105D4541-F2D2-5036-8632-53654B5DDE37

https://zoobank.org/E88548D2-D19F-4C81-9DD9-37471ACF849B

Figs 1–3

##### Type material.

***Holotype.*** Chile • 1♀; Región del Maule, Cauquenes, El Secreto de Pilén; 35°59′1″S, 72°28′21″W; 1, 370 m alt.; 09 Sep–09 Oct 2024; Malaise trap; R.O. Araujo, D.G. Pádua & B. Cortés-Rivas leg.; (MNNC).

##### Type locality.

Chile: Región del Maule, Cauquenes, El Secreto de Pilén; 35°59′1″S, 72°28′21″W; 1, 370 m alt.

##### Diagnosis.

*Scolomusvalenzuelai* sp. nov. can be distinguished from the other species of the genus by the combination of the following characteristics: 1) clypeus lightly punctate with setae, flat in profile, weakly impressed; 2) postscutellum rounded posteriorly; 3) metapleuron strongly strigose near its ventral margin; 4) lateromedian longitudinal carina elevated, strong until the intersection with the anterior transverse carina, and faintly impressed posteriorly, lateromedian longitudinal carina parallel; 5) area basalis present, well delimited, trapezoidal and smooth; 6) lateral longitudinal carina slightly curved in apical third; 7) fore wing with a lightly petiolate areolet, rhomboidal; 8) wings strongly and entirely infuscate, and pterostigma and all veins dark brown.

**Figures 1–3.**
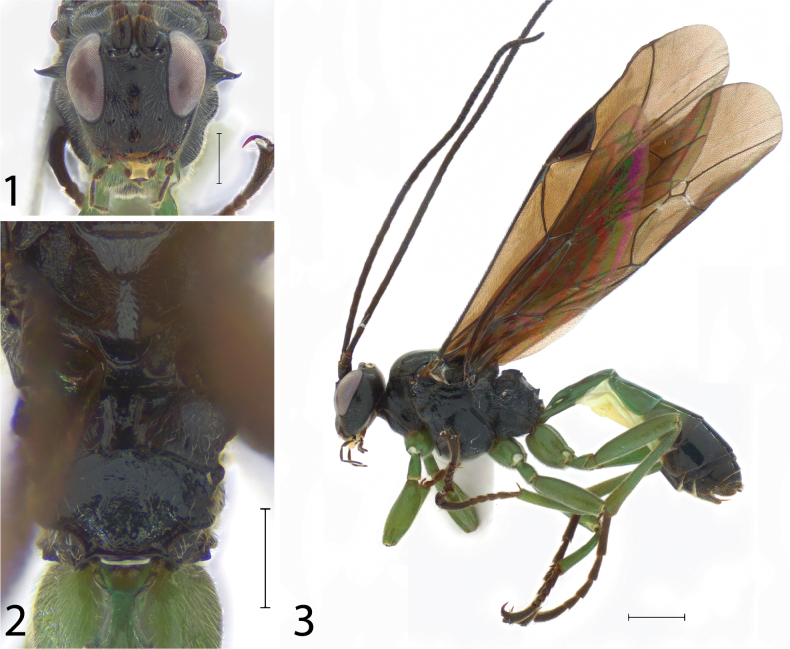
*Scolomusvalenzuelai* Araujo, Pádua & Silva-Santos, sp. nov., holotype female. **1** Head in frontal view **2** propodeum in dorsal view **3** habitus in lateral view. Scale bars: 0.50 mm (**1**, **2**); 1.00 mm (**3**).

##### Description.

Holotype female (Figs 1–3). Body length (without ovipositor): 7.85 mm; antenna length: 6.70 mm; fore wing length: 6.75 mm.

***Head*.** Head polished, with sparse short pubescence. Face narrowly elongate, densely punctate, with setae laterally and dorsally but sparse centrally. Maximum face width about 1.20× as high (measured from base of antennae to base of clypeus) as wide. Anterior tentorial pits visible. Clypeus lightly punctate with setae, flat in profile, weakly impressed, its apical margin truncated, sinuous, 1.40× as wide as high. Labrum slightly visible even with mandibles closed. Malar space 1.25× as long as basal mandibular width; mandible relatively stout, mandible basally and centrally punctate, with setae. Mandible bidentate, teeth smooth, upper tooth longer than lower tooth. Upper tooth 3.35× as long as basally wide. Frons without distinct punctures. Inner orbits almost parallel, very slightly indented opposite antennal socket. Stemmaticum slightly convex. Posterior ocellus separated from eye about 0.95× its maximum diameter. Distance between posterior ocelli 1.15× the maximum diameter of posterior ocellus. Vertex shiny, without punctures. Temple shiny with setiferous punctures; temples almost parallel behind eye. Antenna with 36 flagellomeres; first flagellomere 3.85× as long as apically wide.

***Mesosoma*.** Mesosoma polished, with very short whitish setae, longer on propodeum. Pronotum with very small punctures with setae, smoother laterally. Mesoscutum slightly convex dorsally, strongly carinate, especially posteriorly; notaulus faintly impressed anteriorly. Mesoscutum polished, with very small, dense, and evenly distributed punctures. Scuto-scutellar groove very deep. Scutellum convex in profile, with strong anterolateral carinae. Postscutellum rounded posteriorly. Scutellum and postscutellum polished, elevated, with very small and dense punctures. Subtegular ridge produced into a sharp, curved spine. Mesopleuron polished, with small, dense punctures with setae on dorsal half anterior to speculum and on ventral half; speculum polished and entirely smooth. Epicnemial carina complete, strong, elevated, reaching anteroventral margin of mesopleuron. Sternaulus indistinct. Posterior transverse carina of mesosternum complete, medially strongly excised. Posterior margin of mesosternum expanded and upcurved, producing into a small lobe. Metapleuron polished, with many small punctures, strongly strigose and convex near its ventral margin, about 1.15× as long as height. Submetapleural carina complete, strong, produced anteriorly and posteriorly into a small lobe. Propodeal spiracle circular, almost connected to laterolongitudinal carina. Propodeum shiny, in dorsal view about 1.10× as medially wide as long. Anterior transverse carina complete, medially lightly excised. Laterolongitudinal carina complete, strong, explanate above spiracles, elevated at intersection with anterior transverse carina and with posterior transverse carinae, forming apophyses just after the last-mentioned intersection. Posterior transverse carinae absent medially, between apophyses; lateromedian longitudinal carina elevated, strong until the intersection with the anterior transverse carina, and faintly impressed posteriorly; lateromedian longitudinal carinae parallel. Area basalis present, well delimited, trapezoidal, and smooth. Area externa shiny and smooth. Lateral longitudinal carina slightly curved in apical third. Coxae shiny, punctate with setae well distributed throughout. Hind femur about 5.35× as long as its maximum height and about 0.90× as long as hind tibia. Tarsal claws large, longer than arolium. Fore wing with large pterostigma and a lightly petiolate areolet, rhomboidal. Vein 1*cu-a* inclivous, lightly postfurcal relative to M&RS. Distal abscissa of *Rs* very slightly sinuate. Abscissa of CU present and touching wing posterior margin. CU strongly inclivous, cu-a reclivous.

***Metasoma*.** Metasoma polished, with very short and relatively sparse setae. Tergite I about 1.20× as long as posteriorly wide. Spiracle near its center, smooth, with isolated setiferous punctures. Dorsolateral carina of tergite I absent. Postpetiole 4.35× as long as maximum width. Glymma deep, seemingly with thin membrane between both sides. Tergal-sternal suture of first metasomal segment complete and strong. Tergite II 2.00× as long as its height (lateral view). Thyridium not discernible. Tergites III–VII similarly sculptured. Hypopygium large and triangular in lateral view, 2.85× as long as wide. Ovipositor short, needle-shaped, 5.00× as long as basal width.

***Color*.** Predominantly black and turquoise-green. Head black; antenna, basal half of mandible, and palpi brownish black. Mesosoma entirely black. Tegula black; legs with all coxae, trochanters, femurs, and tibia turquoise-green; all trochantelli brownish black. Wings strongly and entirely infuscate; pterostigma and all veins dark brown. Metasoma turquoise green, with posterior margin of tergites III, T-shaped mark extending from the apical margin to the center of tergite III; tergites VI onwards black. Ovipositor sheath black, ovipositor yellowish red. Body covered by silvery pubescence.

**Male.** Unknown.

##### Etymology.

*Scolomusvalenzuelai* is named in honor of Pablo Valenzuela, a distinguished Chilean biochemist whose pioneering contributions to biotechnology and molecular biology have been profound in both scientific research and public health. Dr Valenzuela’s crucial role in the development of the hepatitis B vaccine, the identification of the hepatitis C virus, and Chile’s genomic research advancement has significantly shaped the country’s biomedical innovation. His work fosters a legacy of scientific excellence and technological progress. By dedicating this species to him, we recognize science’s debt to his invaluable contributions and his enduring influence on future generations’ research. The species epithet, *valenzuelai*, is to be treated as a noun in genitive case.

##### Distribution.

Known only from the type locality (Fig. 7).

##### Biology.

Unknown.

##### Comments.

*Scolomusvalenzuelai* sp. nov. is most similar to the South American *S.magellanicus* and *S.viridis* by the stout mandible; the subtegular ridge produced into a sharp, curved spine; a deep groove between the propodeum and metanotum; and predominantly green coloration. The new species can be differentiated from these species by (1) the lateromedian longitudinal carina, which is strong and elevated until the intersection with the anterior transverse carina, then becoming faintly impressed posteriorly; the parallel lateromedian longitudinal carina (vs. lateromedian longitudinal carina converging posteriorly, generating a reduced, triangular area basalis in *S.magellanicus*; and lateromedian longitudinal carina strongly impressed posteriorly in *S.viridis*); (2) fore wing with a lightly petiolate areolet, rhomboidal (vs. a pentagonal areolet, with vein 2rs-m complete and 3rs-m partially complete and both independently touching RS); 3) the strongly and entirely infuscate wings, with the pterostigma and all veins dark brown (vs. wings with pterostigma and all veins brown, hyaline to lightly infuscate in *S.magellanicus* and lightly infuscate in *S.viridis*); 4) the posteriorly rounded postscutellum (vs. posteriorly straight in *S.magellanicus* and *S.viridis*). Additionally, *S.valenzuelai* sp. nov. differs from *S.magellanicus* by having the lateral longitudinal carina slightly curved in its apical third (vs. straight in its apical third); the hypopygium large, 2.85× as long as wide (vs. hypopygium 1.70× as long as wide); and differs from *S.viridis* due the absence of the superomedia area (vs. present, although open posteriorly).

#### ﻿ Scolomus magellanicus

Taxon classificationAnimaliaHymenopteraIchneumonidae

Walkley, 1962

7C255EAD-DE37-5554-885D-864780657DCF

Figs 4–6

##### Examined material.

Chile • ***Holotype***: ♀; Magellanes, El Ganso; 14 Feb 1952; Maria Etcheverry C. leg.; NMNH 001 (digital images examined) • 1♀; Magallanes, El Canelo; 8 Mar 1969; L. Peña leg.; MNNC • 1♀; Magallanes, Punta Arenas; 15 Mar 1969; L. Peña leg.; MNNC • 1♀; Nuble Prov., Refugio Las Cabras, Cord. Chillan; 1500 m alt.; L.E. Peña leg.; MNNC • 1♀; Chiloé Prov., Dalcahue; 1–30 Jan 2022; G. Barriga leg.; Malaise trap; LEGA-UCM 001.

##### Diagnosis.

*Scolomusmagellanicus* can be distinguished from the other species of the genus by the combination of the following characteristics: 1) clypeus lightly punctate with setae, uniformly distributed, moderately impressed and convex in profile; 2) postscutellum straight posteriorly; 3) metapleuron moderately strigose near its ventral margin; 4) lateromedian longitudinal carina posteriorly converging, generating a reduced and triangular area basalis, and faintly impressed posteriorly to the anterior transverse carina; 5) lateral longitudinal carina straight in apical third; 6) fore wing with a pentagonal areolet, with vein 2rs-m complete and 3rs-m partially complete (faintly touching vein M), and both touching RS independently; 7) wings hyaline or lightly infuscate, pterostigma and all veins brown.

##### Description.

**Figures 4–6.**
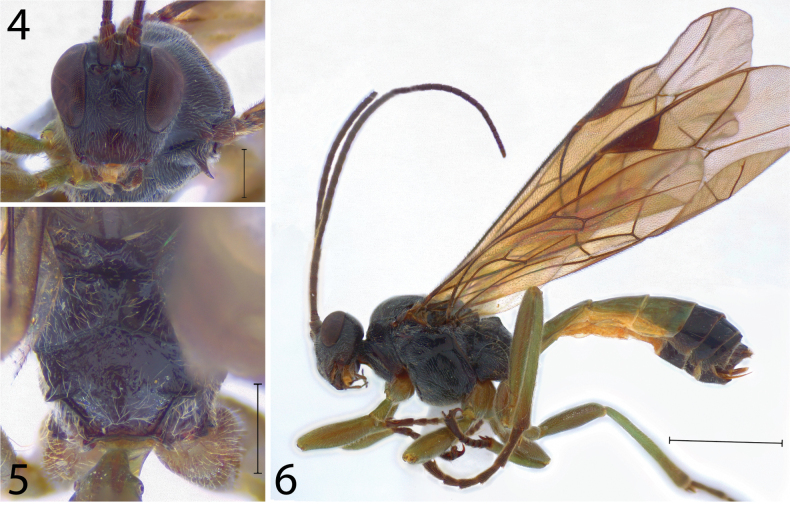
*Scolomusmagellanicus* Walkley, 1962, female. **4** Head in frontal view **5** propodeum in dorsal view **6** habitus in lateral view. Scale bars: 0.50 mm (**4**, **5**); 1.00 mm (**6**).

Female (Figs 4–6). Approximate body length (without ovipositor): 7.80 mm; antenna length: 7.00 mm; fore wing length: 7.60 mm.

***Head*.** Head polished, with sparse, short pubescence. Face narrowly elongate, densely punctate, with small, uniformly distributed setiferous punctures. Maximum width of face about 1.40× width (measured from base of antennae to base of clypeus). Anterior tentorial pits visible. Clypeus lightly punctate, evenly covered with setae, moderately impressed, convex in profile, its apical margin truncate and sinuous, 1.20× as wide as high. Labrum slightly visible with mandibles closed. Malar space 1.45× as long as basal mandibular width. Mandible relatively stout, basally and centrally punctate with setae. Mandible bidentate, with teeth smooth; upper tooth 3.00× as long as its width at base, and slightly longer than lower tooth. Frons without distinct punctures. Inner orbits almost parallel, very slightly indented opposite antennal socket. Stemmaticum slightly convex. Posterior ocellus separated from eye about 0.95× its maximum diameter. Distance between posterior ocelli 1.15× maximum diameter of posterior ocellus. Vertex shiny, without punctures. Temple shiny with setiferous punctures; temples almost parallel behind eye. Antenna with 37 flagellomeres, first flagellomere 3.80× as long as width at apex.

***Mesosoma*.** Mesosoma polished, with very short setae, longer in propodeum. Pronotum with very small, setiferous punctures but laterally smoother. Mesoscutum slightly convex dorsally, strongly carinate, especially posteriorly; notaulus faintly impressed anteriorly; mesoscutum polished, with very small, dense, evenly distributed punctures. Scuto-scutellar groove very deep. Scutellum convex in profile, with strong anterolateral carinae. Postscutellum straight posteriorly. Scutellum and postscutellum polished, elevated, with very small, dense punctures. Subtegular ridge produced into a sharp, curved spine. Mesopleuron polished, with small, dense punctures with setae on dorsal half anterior to speculum and on ventral half. Speculum polished and entirely smooth. Epicnemial carina complete, strong, elevated, reaching anteroventral margin of mesopleuron. Sternaulus indistinct. Posterior transverse carina of mesosternum complete and medially, strongly excised. Posterior margin of mesosternum expanded and upcurved, produced into a small lobe. Metapleuron polished, moderately strigose and convex near its ventral margin, about 1.30× as long as height; metapleuron with many small punctures with long setae. Submetapleural carina complete, strong, produced anteriorly and posteriorly into a small lobe. Propodeal spiracle circular, almost connected to laterolongitudinal carina. Propodeum shiny, in dorsal view about 1.20× as medially wide as long. Anterior transverse carina complete, medially lightly excised. Laterolongitudinal carina complete, strong, explanate above spiracles, elevated at intersection with anterior transverse carina and with posterior transverse carina, forming apophyses just after this intersection. Posterior transverse carina absent medially, between apophyses. Lateromedian longitudinal carina elevated until the intersection with the anterior transverse carina and faintly impressed posteriorly. Lateromedian longitudinal carina converging posteriorly, generating a reduced and triangular shaped area basalis. Area externa shiny and smooth. Lateral longitudinal carina straight in apical third. Coxae shiny, punctate with well-distributed setae throughout. Hind femur about 5.50× as long as its maximum height and about 0.75× as long as hind tibia. Tarsal claws large, longer than arolium. Fore wing with large pterostigma and a pentagonal areolet, with vein 2rs-m complete and 3rs-m partially complete (faintly touching vein M), both touching RS independently. Vein 1*cu-a* inclivous, lightly postfurcal relative to M&RS. Distal abscissa of *Rs* very slightly sinuate. Abscissa of CU present and touching wing posterior margin. CU strongly inclivous; cu-a reclivous.

***Metasoma*.** Metasoma polished, with very short and relatively sparse setae. Tergite I about 1.80× as long as posteriorly wide. Spiracle near its center, smooth, with isolated setiferous punctures. Dorsoleteral carina of tergite I absent. Postpetiole 3.60× as long as maximum width. Glymma deep, seemingly with thin membrane between both sides. Tergal-sternal suture of first metasomal segment complete and strong. Tergite II 2.90× as long as its height (lateral view). Thyridium not discernible. Tergites III–VII similarly sculptured. Hypopygium triangular in lateral view, 1.70× as long as wide. Ovipositor short, needle-shaped, 5.90× as long as basal width.

***Color*.** Predominantly black and pale green. Head black; antenna, clypeus, basal half of mandible, and palpi dark brown. Mesosoma entirely black; tegula black; legs with all coxae, trochanters, femurs, and tibiae pale green; all trochantelli brown. Wings lightly infuscate; pterostigma and all veins brown. Metasoma pale green, with tergites VI onwards black; Ovipositor sheath black; ovipositor yellowish red. Body covered by silvery pubescence.

##### Variation.

Approximate body length (without ovipositor): 7.80–9.45 mm; fore wing length 7.60–9.50 mm. Antenna with 37–39 flagellomeres. Clypeus dark brown to reddish brown. Wings hyaline to lightly infuscate.

##### Distribution.

**Figure 7.**
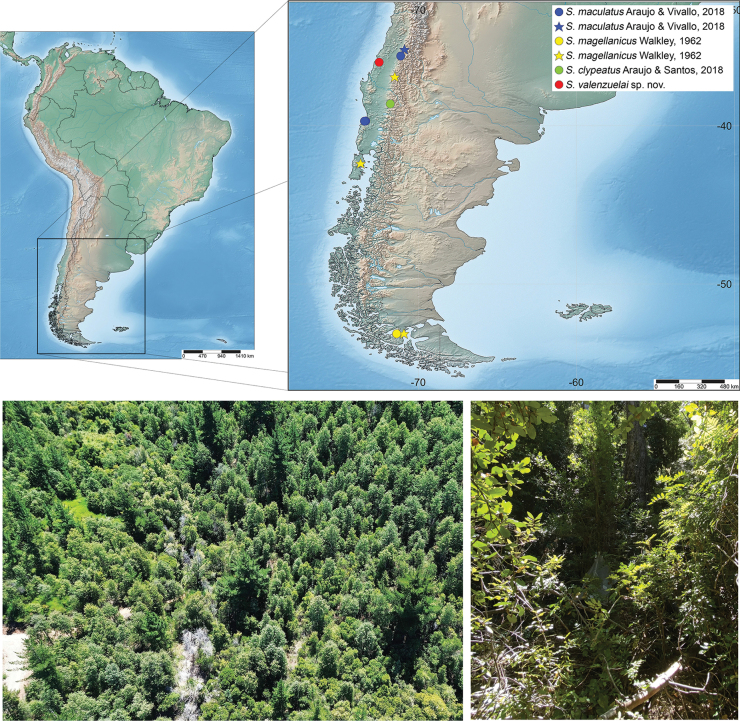
Distribution of *Scolomus* in Chile. Blue circle: previous distribution of *S.maculatus* Araujo & Vivallo, 2018; blue star: new distribution record of *S.maculatus*; yellow circle: previous distribution of *S.magellanicus* Walkley, 1962; yellow star: new distribution record of *S.magellanicus*; green circle: distribution of *S.clypeatus* Araujo & Vivallo, 2018; red circle: distribution of *S.valenzuelai* Araujo, Pádua & Silva-Santos, sp. nov. Imagens bellow showing the vegetation and the Malaise trap in the field.

Chile: Región de Los Lagos: Chiloé (Dalcahue*); Región del Ñuble: Pinto (Refugio Las Cabras*); Región Magallanes y la Antartica Chilena: Magallanes (El Canelo*, El Ganso, and Punta Arenas*) (Fig. 7).

#### ﻿ Scolomus maculatus

Taxon classificationAnimaliaHymenopteraIchneumonidae

Araujo & Vivallo, 2018

88B35320-EE6E-5AA8-B5DB-BBEDCC659B30

##### Examined material.

Chile • ***Holotype***: ♀; Región Los Ríos, Valdivia, Parque Oncol, Cordillera de la Costa, Bosque Siempreverde; 39°42′10″S, 73°18′31″W; 2, 493 m alt.; 06–20 Mar 2007; Cecilia Ruiz et al. leg.; Malaise trap; MNNC (digital images examined) • 1♀; Curicó, 20km E Potrero Grande • 1♂; Región Los Ríos, Valdivia, Parque Oncol, Cordillera de la Costa, Bosque Siempreverde; 39°42′0.48″S, 73°19′36.54″W; 1, 569 m alt.; 20 Mar–04 Apr 2007; Cecilia Ruiz et al. leg.; Malaise trap; UACh 002 • 1♂; Región Los Ríos, Valdivia, Reserva Privada Punta Curiñanco, Ecorregión Valdiviana, Bosque Siempreverde; 5602646N, 636889E; 06–20 Mar 2007; Cecilia Ruiz et al. leg.; Malaise trap (trampa A, curi–3(?)); UACh 003 • 2♀, 2♂; Región Los Ríos, Valdivia, Reserva Privada Punta Curiñanco, Ecorregión Valdiviana, Bosque Siempreverde; 5602646N, 636889E; 18 Apr–02 May 2007; Cecilia Ruiz et al. leg.; Malaise trap (trampa A, curi–3(?)); UACh 004 • 1♀; Región Los Ríos, Valdivia, Parque Oncol, Cordillera de la Costa, Bosque Siempreverde; 39°42′0.48″S, 73°19′36.54″W; 1, 569 m alt.; 01–20 Feb 2007; Cecilia Ruiz et al. leg.; Malaise trap; UACh 005 • 1♀, 1♂; Región Los Ríos, Valdivia, Parque Oncol, Cordillera de la Costa, Bosque Siempreverde; 39°42′0.48″S, 73°19′36.54″W; 1, 569 m alt.; 05–19 Jan 2007; Cecilia Ruiz et al. leg.; Malaise trap; UACh 006.

##### Diagnosis.

See Araujo et. al. (2018).

##### Distribution.

Chile: Región de Los Ríos, Valdivia, (Parque Oncol and Reserva Punta Curiñanco). Región del Maule: Talca (Altos de Vilches and Curicó*) (Fig. 7).

## ﻿Discussion

The known Argentine and Chilean *Scolomus* species have been described based on a single specimen or only a few specimens (e.g., Townes and Townes 1950; Walkley 1962; Araujo et al. 2018). In this study, approximately 25,000 Chilean Darwin wasp specimens were examined in the LEGA-UCM, MNNC, and UACh collections. Among these, only 15 specimens were identified as belonging to *Scolomus*: 10 specimens of *S.maculatus*, four *S.magellanicus*, and one specimen, which is described here as a new species. The scarcity of specimens underscores the rarity of this genus in entomological collections. This limited representation suggests that *Scolomus* may have a restricted distribution in the Andean biogeographic zone (sensu Morrone 2015), low population densities, or specific ecological requirements that hinder its collection and study.

## Supplementary Material

XML Treatment for Scolomus

XML Treatment for Scolomus valenzuelai

XML Treatment for Scolomus magellanicus

XML Treatment for Scolomus maculatus

## ﻿Additional information

### Author ORCIDs

Rodrigo O. Araujo https://orcid.org/0000-0002-9438-3238

Isamara Silva-Santos https://orcid.org/0000-0001-8802-6720

Andrés Moreira-Muñoz https://orcid.org/0000-0002-9136-1391

Cristian Montalva https://orcid.org/0000-0002-8357-774X

Diego G. Pádua https://orcid.org/0000-0001-5061-2978

### Data availability

All of the data that support the findings of this study are available in the main text.
